# Padel, pickleball and wellbeing: a systematic review

**DOI:** 10.3389/fpsyg.2025.1614448

**Published:** 2025-07-29

**Authors:** Lena Lauxtermann, Brendon Stubbs

**Affiliations:** ^1^Department of Sport and Exercise Physiology, Centre for Sport Science and University Sports, University of Vienna, Vienna, Austria; ^2^Department of Psychological Medicine, Institute of Psychiatry, Psychology, and Neuroscience (IoPPN), King’s College London, London, United Kingdom

**Keywords:** mental health, wellbeing, tennis, paddle, pickleball

## Abstract

**Introduction:**

Physical activity benefits mental health, yet the effects of emerging sports like padel and pickleball are understudied despite their accessibility and growth—300,000 amateur padel players worldwide and a 223.5% rise in U. S. pickleball players (2020–2023). This systematic review examines their association with mental health, wellbeing, and mental fatigue.

**Methods:**

An electronic search of Medline, PsycINFO, and Embase (inception to October 8, 2024; PROSPERO CRD42024594743) identified quantitative and observational studies on padel or pickleball participation and mental health or wellbeing outcomes.

**Results:**

Fourteen of 71 studies (*n =* 1,403) were included. Pickleball enhances wellbeing, life satisfaction (*p* < 0.05), happiness (r = 0.263, *p* < 0.001), depression (r = −0.23, *p* < 0.01), and social integration, especially in older adults. In padel, higher-level and match-winning players show increased self-confidence and reduced somatic anxiety; pre-competition anxiety rises, varies by gender and score, and is lower than in tennis. Mental fatigue increases with successive padel games, impairing accuracy (*p* = 0.05) and linking to elevated motivation.

**Conclusion:**

Mental health research on padel and pickleball has expanded, revealing distinct areas of focus. Padel studies primarily target performance aspects like self-confidence, anxiety, and mental fatigue, while pickleball research highlights wellbeing gains in life satisfaction, happiness, and social integration. However, gaps remain, particularly regarding the exploration of wellbeing in padel and performance-related mental health (e.g., anxiety, fatigue) in pickleball across diverse age groups to address these gaps. Future studies should address these gaps and consider diverse age groups to provide a more comprehensive understanding.

**Systematic review registration:**

PROSPERO: The Unique Identifier is CRD42024594743, and the publicly accessible URL is https://www.crd.york.ac.uk/prospero/display_record.php?ID=CRD42024594743.

## 1 Introduction

Over 40% of the global population fails to meet physical activity guidelines (WHO, 2024; Bull et al., 2020), missing established benefits to cognitive, mental, and physical health (Lubans et al., 2016; Penedo and Dahn, 2005). Addressing this inactivity crisis requires innovative, inclusive, and enjoyable exercise options. Padel and pickleball, emerging racket sports, are gaining traction, with 300,000 amateur and 600,000 federated padel players across 130 + countries (Fip, 2024) and a 223.5% increase in U. S. pickleball players from 2020 to 2023 (SFIA Pickleheads, 2024). Interest in research is also rising (García-Giménez et al., 2022), as padel has been identified as a driver of wellbeing and sustainable local growth (Nicosia and Privitera, 2024).

Their popularity stems from low technical barriers (Sánchez-Alcaraz and Courel-Ibáñez, 2022), social engagement (Smith et al., 2017), enjoyment (Riffee et al., 2023), affordability (Wray et al., 2021), age inclusivity (Cerezuela et al., 2023), and outdoor settings enhancing wellbeing (Demeco et al., 2022). Padel peaks among ages 35–43 (Courel Ibáñez et al., 2017), while pickleball attracts mostly those over 50 (Cerezuela et al., 2023), though younger players are increasing (USA Pickleball Association, 2022). Intensity varies from low to vigorous (Casper et al., 2023; De La Fuente et al., 2023), broadening accessibility.

Padel involves quick changes of direction, short sprints and precise shots on a fenced-in plastic field made of glass and metal (20 × 10 m) (Fip, 2017). The ability to bounce the ball off the walls introduces a unique tactical element and creates a dynamic and fast-paced style of play without increasing physical intensity (Gea García et al., 2021; Courel-Ibáñez et al., 2019). Recently, it has been emphasized that padel is mentally and physically demanding, as it is a cognitively demanding activity where players must make decisions in a complex environment under time pressure (Díaz-García et al., 2021a; Díaz-García et al., 2024).

In contrast, pickleball is played on a smaller court (13.41 × 6.1 m) using a perforated plastic ball that reaches lower speeds compared to other racket sports (Kim et al., 2021). This setup reduces physical intensity while emphasizing social interaction and strategic play over technical gestures (Michael and Webster, 2020).

Studies have explored physical fitness, injury risk (Demeco et al., 2022), attentional focus (Starzak et al., 2024), and performance metrics (e.g., Conde-Ripoll et al., 2024c, Pradas et al., 2021b), alongside cardiovascular (Courel-Ibáñez et al., 2018) and brain health benefits (Pradas et al., 2021a). Yet, mental health and wellbeing remain underexplored, despite evidence of mental fatigue in racket sports affecting performance (Díaz-García et al., 2024; Le Mansec et al., 2018) and motivation’s role in fatigue tolerance (McMorris, 2020). Current literature prioritizes physical outcomes, particularly in padel (Sabe et al., 2022), leaving a gap in mental health research.

Thus, this systematic review investigates the association between regular padel and pickleball play and mental health, wellbeing, and mental fatigue.

## 2 Methods

This systematic review was registered in the International Prospective Register of Systematic Reviews (PROSPERO: CRD42024594743) and conducted in accordance with the Prisma Statement (Page et al., 2021).

### 2.1 Search strategy and selection criteria

An electronic search was conducted in the following databases Medline(r), Psychinfo, Embase for any quantitative study (interventions, controlled trials, pre and post-test studies) and observational studies (cohort or cross-sectional studies) that measured the relationship between padel or pickleball participation and wellbeing or mental health in humans. Studies comparing an intervention of padel or pickleball and another racket sport (e.g., badminton, tennis), any other sport or no sport were included. Only articles published in English and German were considered.

The search terms used were (padel or pickleball) and mental health (wellbeing or mental or stress or eustress or distress or depression or mental disorder or emotional functioning or anxiety or self-confidence or lifestyle or social or quality of life or emotion). Upon conduction of the searches, duplicates were removed electronically. The reference lists of eligible articles from all existing reviews investigating the mental health or wellbeing effects of padel and pickleball were screened to identify potentially eligible articles. One author undertook the abstract and full text screening, and these were independently verified by a second author. If a paper was not available in full text but was considered potentially suitable, the corresponding authors were contacted (*n =* 1). A PRISMA flowchart was created to document the study selection process.

### 2.2 Primary outcome

The primary outcome was the mental health and wellbeing effects of padel and pickleball. Although there is no universally accepted definition of wellbeing, nor a universally accepted or standardized tool for assessing wellbeing, it is associated with a positive state of mind (Linton et al., 2016). Mental health can be understood as a person’s ability to think and act in a way that promotes their own wellbeing and helps with stress management and awareness of personal and social boundaries (Encyclopædia, 2024).

### 2.3 Secondary outcome

Any other health-related outcomes were considered including:

1. Perception of stress (e.g., eustress, distress).
2. Lifestyle changes (e.g., changes in diet, physical activity level).
3. Social outcomes (e.g., communication, fun, social contact).
4. Quality of life (measured by any established questionnaires).
5. Emotional functioning (e.g., self-confidence, anxiety).

### 2.4 Data extraction

Data were extracted by one author and independently verified by a second author. Inter-rater reliability was first determined before the screening process by randomly selecting 20% of all trials. Both reviewers checked and compared their decisions based on these studies. Only if a match of 95% or more was found, both reviewers independently performed abstract and title screening. Data from the studies included were extracted by the reviewers using a standardized Excel form developed for the review. This included information on publication year and country, study design, study setting, participant and intervention characteristics, follow-up details, primary and secondary outcomes and confounders. If data were missing or details were unclear, the corresponding author of the study was contacted for verification.

### 2.5 Study quality assessment

The quality assessment was carried out by one independent assessors using the PEDro scales for controlled trials (Page et al., 2021) and the Newcastle Ottawa scale for observational studies (Wells et al., 2000). These were reviewed by a second author. While a maximum of 10 points can be achieved on the PEDro scale (Page et al., 2021), 9 stars is the maximum on the Newcastle Ottawa scale (Wells et al., 2000). The quality of the study assessed using the PEDro scale was categorized as follows: “excellent” (9–10), “good” (6–8), “fair” (4–5), “poor” (0–3). The classification of the quality of the NOS is not as standardized as the PEDro Scale, but in the literature 7–9 points are often considered high quality, 4–6 points as moderate quality and anything <4 points as poor quality with a high risk of bias.

### 2.6 Data analysis

The data captured for each study was summarized in a narrative synthesis. For intervention studies, any quantitative data including the mean, mean differences, effect sizes and 95% Confidence intervals (CI) were reported as well as any inferential data from observational studies that report an association between padel or pickleball and mental health or wellbeing and the adjusted confounders. To improve the comparability of the studies, the tables of characteristics of the included studies were categorized into the following three thematic areas: Mental Health, Anxiety and Mental Fatigue. The results are presented in three columns in Table 1 for clarity.

**Table 1:** Results of the included studies (*n =* 14).

Author (publication year)	Sport, level, investigation	Mental health	Mental fatigue	Anxiety
Casper et al. (2021)	Pickleball, recreational Seniors, pre-post-COVID-19	Limited where and how seniors get PA, but many are still active Socializing ↓ Perceived physical health ↔ Mental health ↓, Loneliness ↑ Life satisfaction ↓		
Castillo-Rodriguez et al. (2022)	Padel, different level (C1-C3)	SC ↑ in higher category players than lower category players Category, BMI and experience predicted 82% of the variance of SC		SA ↓ and CA ↑ in higher category players than lower category players
Conde-Ripoll et al. (2023)	Padel, high level	SC was not affected by ranking, seed, result or round ↔		CA, SA, STA not affected by ranking, result↔ CA ↓ for seeded players STA ↓ for winners in M1 STA ↓ in M2 than in M1 Lower ranked players: SA and STA ↑ in R16 than M2 STA ↑ in M1 than M3
Conde-Ripoll et al. (2024a)	Padel, high level	SC ↑ pre-competition than training (for left-side, higher-ranked, lower-ranked, match winning players)		SA ↑ pre-competition than training (for left-side, higher-ranked, match winning players) SA and STA ↑ right-sided than left-sided players before training matches
Conde-Ripoll et al. (2024b)	Padel, high level	SC ↓ for losing players (pre-post-match) SC ↓ for losing players than winning players		CA, SA, STA ↑ for losing players and STA ↑ for winning players (pre-post match)
Díaz-García et al. (2021b)	Padel, Elite Youth, Constrained vs. Unconstrained matches	Intrinsic and extrinsic motivation ↑ prior constrained matches Identified regulation ↔ amotivation ↓ prior constrained matches	Mental load ↑ after constrained matches Mental fatigue ↑ after constrained matches	
Díaz-García et al. (2021a)	Padel, professional, consecutive matches	motivation ↓ (pre-post match) motivation ↔ in M1 and M2 Performance satisfaction ↓ ➔ motivation ↓	MF ↑ (pre-post match) MF ↑↑ in M1 than M2 Performance satisfaction ↑↓ ➔ MF ↔	
Díaz-García et al. (2024)	Padel, Elite Youth, Mental fatigue condition (30-min incongruent Stroop task) vs. control		MF ↑ after mental-fatigue-condition MF ↔ after control-condition	
Heo et al. (2018)	Pickleball, competitive older players	Commitment to serious leisure ↑ ➔ depression level ↓ Gender and serious leisure* were significant predictors of depression		
Kim et al. (2021)	Pickleball, competitive older players	PA-Participation × general happiness and social capital (community participation, feeling of trust and safety, neighborhood connection) + PA-Participation was a significant predictor (adjusted R^2^ = 0.113) of general happiness		
Riffee et al. (2023)	Pickleball, missing information	Motivation through: enjoyment (4.84), physical health (4.6), social health (4.43), psychological health (4.38), and competition (4.38) Benefits of playing Pickleball: social health (4.68), enjoyment (4.56), and physical health (4.6) Strong relationships between motivations and perceived benefits regarding physical activity within the concepts of psychological health (r = 0.74) and enjoyment (r = 0.71)		
Rodríguez-Cayetano et al. (2022)	Padel and Tennis players, competitive Youth	SC ↑ in padel players than tennis players SC ↔ in girls and boys SC ↓ in senior players than in U14 and U16 SC ↑ in female padel than female tennis players		CA and SA ↔ in padel and tennis players CA ↔ in girls and boys SA ↑ in boys CA and SA ↑ in senior players than in U14 and U16 SA ↑ in senior padel than tennis players CA ↓ in female padel than female tennis players SA ↑ in male padel than male tennis players In all cases: significant positive correlation between CA and SA and a negative correlation between CA and SA with SC
Ryu et al. (2018)	Pickleball, high level older players	Conscientiousness, serious leisure, and openness to experience were significant predictors of eudaimonic wellbeing neuroticism was negatively associated with eudaimonic wellbeing		
Ryu et al. (2022)	Pickleball, high level older players (different age groups: 50–59,60–69, 70+)	Life satisfaction ↑ in oldest age group (70+) Optimism and social integration ↔ between age groups Social integration ↑ in female players Optimism and life satisfaction ↔ between gender		

↑, increase; ↓, decrease; ↔, no significant differences; CA, cognitive anxiety; MF, mental fatigue; PA, physical activity; SA, somatic anxiety; SC, self-confidence; STA, state anxiety; M1, Match one; M2, Match two; +, positive association; CA, cognitive anxiety; *, after controlling for age; gender and education.

## 3 Results

**Figure 1:**
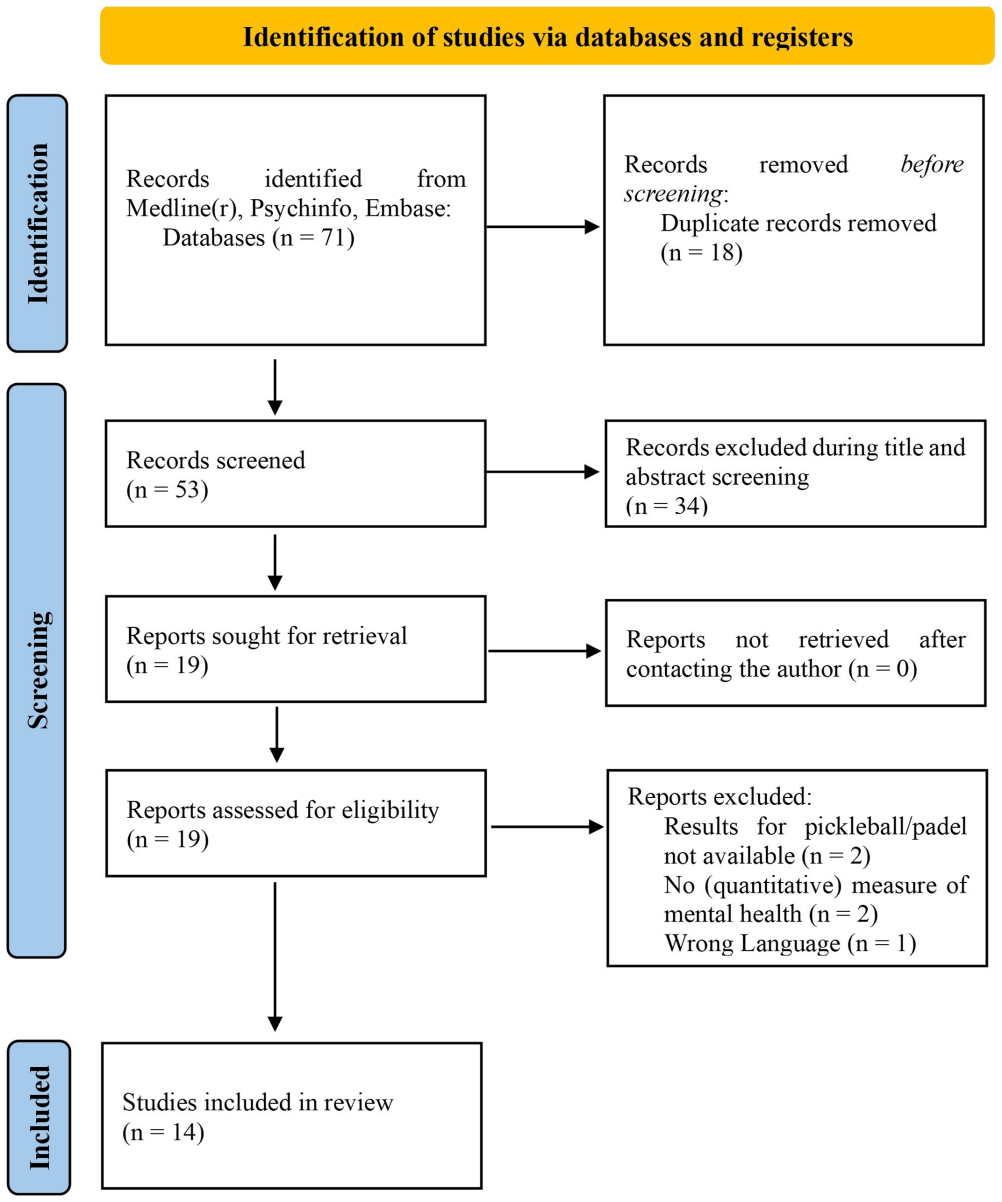
PRISMA 2020 flowchart for study selection. Abbreviation: PRISMA, Preferred Reporting Items for Systematic Reviews and Meta-Analyses. Adapted from Page et al. (2021).

The search results are shown in Figure 1. Of the 71 records, 14 studies met the inclusion criteria.

### 3.1 Overview of studies

A total of 1,403 participants were included in the review. A total of 8 studies assessed the mental health effects of Padel (*n =* 538) with lower average numbers of test subjects (∅ = 67) while the influence of Pickleball on mental health was evaluated in 6 (*n =* 865) studies with on average more than twice as many participants (∅ = 144). Women accounted for 42% of those analyzed. There was no age limit, so the mean age of the youngest group of participants was 15.40 (± 3.43) years, while the oldest participants were over 80 years old. The participants are mostly professional and high level players (*n =* 676) or competitive athletes (688), with smaller proportions of recreational players (*n =* 36) or missing data (*n =* 103). The included studies were conducted in only three countries, mainly the USA (*n =* 6), Spain (*n =* 5) and Finland (*n =* 3) between 2018 and 2024. Characteristics of selected studies are shown in Tables 2–4.

**Table 2:** Studies that have investigated the mental health effects of padel or pickleball (*n =* 6).

Author (publication year)	Number of participants Participant’s characteristics (age, gender)	Intervention type	Mental health outcome	Secondary outcomes
Casper et al. (2021)	*n =* 36 Recreational Pickleball players (>65 years; f: 16; m: 20)	Observational study (Pre- and post-study COVID-19)	Mental health (SF-12 Health survey)	Social connections Loneliness (ULS-4) Life satisfaction (5-item Satisfaction with Life Scale) Physical activity
Heo et al. (2018)	*n =* 153 Competitive Pickleball players (>50 years; f: 90; m: 63)	Observational study (Cross-sectional study)		Depression level (MDI) Serious Leisure (SLIM), Optimism (Life orientation test)
Kim et al. (2021)	*n =* 208 Competitive Pickleball players (50–83 years; f: 96; m: 112)	Observational study (Cross-sectional study)		General happiness (Single item from Abdel-Khalek, 2006), Serious Leisure (SLIM) Social capital (9-item from Onyx and Bullen, 2000)
Riffee et al. (2023)	*n =* 65 Pickleball players (>30 years; f: 31; m: 35)	Mixed-Method-Study		Physical activity and Motive (PALMS) Exercise Benefits/Barriers Scale (Victor et al., 2012)
Ryu et al. (2018)	*n =* 153 High level Pickleball players (>50 years; f: 90; m: 63)	Observational study (Cross-sectional study)		Life satisfaction (SWLS) Optimism (LOT) Social integration (Sonnega et al., 2014)
Ryu et al. (2022)	*n =* 250 High level Pickleball players (Mage: 65.11 ± 7.49; *f* = 145; m = 105)	Observational study (Cross-sectional study)	Eudaimonic wellbeing (QEWB)	Serious Leisure (SLIM) Personality (BFI)

SF-12 Health survey, Short-form-health survey; ULS-4, UCLA Loneliness-Scale; SLIM, Serious Leisure Inventory Measures; PALMS, Physical Activity and Leisure Motivation Scale; MDI = Major Depressive Inventory; SWLS = Satisfaction with Life Scale; LOT = Life Orientation Test.

**Table 3:** Studies that have investigated anxiety and self-confidence in padel.

Author (publication year)	Number of participants Participant’s characteristics (age, gender)	Intervention type	Mental health outcome	Secondary outcomes
Castillo-Rodriguez et al. (2022)	*n =* 100 Male padel players with different levels (Mage = 27.6 ± 7.5; m = 100)	Observational study	Self-confidence (CSAI-2R)	Cognitive and somatic anxiety (CSAI-2R)
Conde-Ripoll et al. (2023)	*n =* 28 High level male padel players (no information about age; m = 28)	Observational study	Self-confidence (CSAI-2R)	Cognitive and somatic anxiety (CSAI-2R) State anxiety (STAI-S)
Conde-Ripoll et al. (2024a)	*n =* 10 High level male padel players (Mage = 28.60 ± 4.17; m = 10)	Observational study	Self-confidence (CSAI-2R)	Precompetitive anxiety (CSAI-2R) State anxiety (STAI-S)
Conde-Ripoll et al. (2024b)	*n =* 11 High level male padel players (Mage = 27.91 ± 5.03; m = 11)	Observational study	Self-confidence (CSAI-2R)	Cognitive and somatic anxiety (CSAI-2R) State anxiety (STAI-S) Technical-tactical performance
Rodríguez-Cayetano et al. (2022)	*n =* 291 (all: *N =* 423) Young padel and tennis players (Mage: 15.40* (all); f: 100; m: 191)	Observational study (Cross-sectional study)	Self-confidence (CSAI-2R)	Cognitive and somatic anxiety (CSAI-2R)

CSAI-2R, Competitive State Anxiety Inventory-2; STAI-S, State–Trait Anxiety Inventory.

**Table 4:** Studies that have investigated mental fatigue in padel.

Author (publication year)	Number of participants Participant’s characteristics (age, gender)	Intervention type	Mental health outcome	Secondary outcomes
Díaz-García et al. (2021b)	*n =* 36 Elite Youth Padel Players (fMage = 17.90 ± 3.21; mMage = 17.40 ± 2.16; f: 14; m: 22)	Observational study (pre-post test study)	Mental load (QQML) Mental fatigue (VAS)	Motivation (SIMS) HRV Reaction time (PVT)
Díaz-García et al. (2021a)	*n =* 14 Professional Players (fMage = 21.70 ± 3.85; mMage = 25.56 ± 6.77; f: 5; m: 9)	Observational study	Mental fatigue (VAS)	Motivation (Likert Scale) Reaction time (PVT) Performance Satisfaction
Díaz-García et al. (2024)	*n =* 48 Elite Youth Padel Players (Mage = 18 ± 2, m = 48)	Randomized counterbalanced crossover study	Mental fatigue (VAS)	Reaction time (PVT) Response Inhibition (Stroop Performance) Padel-specific accuracy (from Sánchez-Alcaraz et al., 2016)

QQML, Questionnaire to Quantify Mental Load; VAS, Visual Analogue Scale; SIMS, Situational Motivation Scale; PVT, Psychomotor Vigilance Test.

### 3.2 Study characteristics of the included studies

Of the included studies, all studies are observational (*n =* 14), the majority of which are cross-sectional in design (*n =* 12) and only a small number of which included pre-post tests (*n =* 2).

All six studies categorized in Table 2 were conducted with pickleball players (*n =* 865). With the exception of one study (>30 years) (Riffee et al., 2023), the test subjects were at least 50 years old. In addition to wellbeing (*n =* 1) (Ryu et al., 2022), social aspects like social connectedness, social capital and integration (*n =* 3) (Ryu et al., 2018; Casper et al., 2021; Kim et al., 2021), life satisfaction (*n =* 2) (Casper et al., 2021; Ryu et al., 2018), and serious leisure (*n =* 3) (Ryu et al., 2022; Heo et al., 2018; Kim et al., 2021) were analyzed. Loneliness (Casper et al., 2021) and optimism (Ryu et al., 2018) were each the subject of one study. Physical activity was also analyzed twice (Casper et al., 2021; Riffee et al., 2023), along with the perceived benefits and barriers of the players (Riffee et al., 2023) (Table 5).

**Table 5:** Quality of the studies included assessed by the Newcastle-Ottawa scale (Wells et al., 2000).

Author (publication Year)	Selection (max. 5 points)	Comparability (max. 2 points)	Outcome (max. 3 points)	Total score (max. 9 points)
Casper et al. (2021)	5	0	2	●7
Castillo-Rodriguez et al. (2022)	3	1	2	●6
Conde-Ripoll et al. (2023)	3	0	2	●5
Conde-Ripoll et al. (2024a)	3	0	2	●5
Conde-Ripoll et al. (2024b)	4	0	2	●6
Díaz-García et al. (2021b)	3	0	2	●5
Díaz-García et al. (2021a)	4	0	2	●6
Heo et al. (2018)	3	2	2	●7
Kim et al. (2021)	4	2	2	●8
Riffee et al. (2023)	3	0	2	●5
Rodríguez-Cayetano et al. (2022)	3	0	2	●5
Ryu et al. (2018)	3	2	2	●7
Ryu et al. (2022)	5	2	1	●9

Green indicates high-quality studies (7–9 points), and yellow indicates moderate-quality studies (4–6 points).

All of the studies in the area of anxiety included only padel players. Out of a total of 440 participants, only 100 players, all from one study (Rodríguez-Cayetano et al., 2022), were female. The average age, where mentioned is <29 years in all studies in this area (*n =* 4). All five studies are equally concerned with self-confidence and different forms of anxiety: cognitive anxiety (*n =* 5), somatic anxiety (*n =* 5) and state anxiety (*n =* 3).

The studies included in the area of mental fatigue (*n =* 3) exclusively examined padel players. Approximately one fifth of the 98 participants were female (*n =* 19). Youth players were largely included (*n =* 84). In all cases, mental fatigue was quantified using the visual analogue scale. Reaction time was also measured in all studies, whereas motivation was only measured in two studies (Díaz-García et al., 2021a; Díaz-García et al., 2021b). Mental load (Díaz-García et al., 2021b), performance satisfaction (Díaz-García et al., 2021a) and padel-specific accuracy (Díaz-García et al., 2024) were each assessed once.

For a detailed quality assessment, Tables 1, 6 show the domain-specific risk of bias and the overall risk for all included studies. Nine points represented the maximum score. The risk of bias assessment showed that 5 studies indicated a low risk of bias, while 6 were assessed as having some concerns. No other studies were assessed as having a high risk of bias in the NOS-Classification. The poor performance of the statistical analysis due to the lack of confidence intervals was striking (*n =* 11). Further reductions were mainly in the area of comparability due to lack of control of confounding factors (*n =* 7) and unjustified sample size (*n =* 8). The average quality score was 6 ± 1 points. One study showed moderate quality with a score of 5, as assessed by the PEDro scale (Table 6). For full details, see Supplementary material.

**Table 6:** Quality of the included studies assessed by the PEDro-Scale (Page et al., 2021).

Author (publication year)	Randomization (max. 2 points)	Comparability and blinding (max. 4 points)	Data and analysis (max. 4 points)	Total score (max. 10 points)
Díaz-García et al. (2024)	1	2	2	●5

Yellow indicates moderate-quality studies (4–6 points).

### 3.3 Overview of results

Playing pickleball is positively associated with overall happiness (r = 0.263, *p* < 0.001) and social capital by building a sense of trust, safety and community (r = 0.342, *p* < 0.001; r = 0.303, *p* < 0.001; r = 0.215, *p* < 0.01) (Kim et al., 2021). General happiness was significantly predicted by pickleball participation (adjusted R^2^ = 0.113, *p* < 0.001) but not by community participation among 208 competitive pickleball players (Kim et al., 2021). Integrating regular pickleball games increased life satisfaction with increasing age (<70 years) (*p* < 0.05) (Ryu et al., 2018) and reduced depression (r = −0.23, *p* < 0.01) (Heo et al., 2018). This is confirmed by the restrictions imposed by the COVID-19 pandemic, which led to a decline in mental (*p* = 0.02), but not physical health (*p* = 0.44) (Casper et al., 2021). Increased loneliness (*p* = 0.01) and lower life satisfaction (*p* = 0.01) where found during the pandemic with 88.89% of pickleball players (*n* = 36) socializing less (Casper et al., 2021).

Alongside physical and social health and the competitive aspect, fun is the greatest motivational component of middle-aged pickleball players (*n =* 65), whose motivation correlates strongly with the benefits in psychological health (r = 0.74) and enjoyment (r = 0.71) they derive (Riffee et al., 2023). Ryu et al. (2022) examined the predictors of eudaemonic wellbeing: Conscientiousness (*β* = 0.32, *p* < 0.001) and Openness (*β* = 0.18, *p* < 0.01) have a positive effect, while Neuroticism (*β* = −0.22, *p* < 0.01) has a negative impact on eudaimonic wellbeing in high level senior pickleball players (*n =* 250). Optimistic and life satisfaction were unaffected by gender and age (*p* > 0.05), while social integration was greater for female pickleball players (*p* < 0.01, $\eta_{p}^{2}$ = 0.06) (Ryu et al., 2018).

On the one hand, padel increases self-confidence and motivation; on the other hand, it leads to a drop in performance in consecutive matches due to mental fatigue (Díaz-García et al., 2021a; Díaz-García et al., 2021b; Díaz-García et al., 2024) and can cause different forms of anxiety (Castillo-Rodriguez et al., 2022; Conde-Ripoll et al., 2024b; Conde-Ripoll et al., 2024a; Conde-Ripoll et al., 2023; Rodríguez-Cayetano et al., 2022). However, this depends on the following factors:

The higher the level of the padel player (*n =* 100), the more self-confidence and less somatic anxiety they have (Castillo-Rodriguez et al., 2022). Compared to pressure training, all players have both more somatic anxiety and more self-confidence before matches (Conde-Ripoll et al., 2024a). This is confirmed for left-handed, higher-ranked and match-winning players (Conde-Ripoll et al., 2024a). The increased self-confidence before competitions also applies to lower-ranked players (Conde-Ripoll et al., 2024a). While self-confidence was not influenced by rank, seed, match result or round in another study, seeded players have less cognitive anxiety (Conde-Ripoll et al., 2023). This is not confirmed in another study by Conde-Ripoll et al. (2024a, 2024b) with 11 high level male padel players as losing a match resulted in higher somatic, cognitive and state anxiety and lower self-esteem than winning players. However, state anxiety is higher in winning players after the game (Conde-Ripoll et al., 2024b). In contrast, state anxiety is higher before the first match than before the second, as well as when the ranking is lower in the first round than in the second in 28 high level padel male players (Conde-Ripoll et al., 2023). Compared to tennis players (*n =* 132), padel players (*n =* 291) were found to have more self-confidence and female players also had lower levels of state anxiety (Rodríguez-Cayetano et al., 2022). Conversely, male padel players showed more somatic anxiety than male tennis players and also than female padel players (Rodríguez-Cayetano et al., 2022). Younger players showed more self-confidence and less pre-competitive anxiety in all sports (Rodríguez-Cayetano et al., 2022).

Díaz-García et al. (2021) tested with elite youth players (*n =* 36) whether a constraint in the form of winning a free training session with a professional player had an effect on their mental fatigue. In addition to increased subjective (*p* < 0.001 for VAS) and objective feelings of mental fatigue (*p* < 0.001 for HRV; *p* = 0.04 for reaction time) motivation was increased, too (Díaz-García; et al., 2021). Female (*n =* 5) and male (*n =* 9) players on the World Padel Tour showed significantly greater mental fatigue after matches than before (Díaz-García et al., 2021a). Mental fatigue accumulated so that the values also differed significantly between the 3 matches, with a maximum after match 3, whereas motivation and reaction time only showed significant differences before and after the match, but not between consecutive matches (Díaz-García et al., 2021a). In the crossover study by the same research group, a control condition was contrasted with a 30-min incongruent Stroop test as a mental fatigue condition in elite male youth players (*n =* 48) (Díaz-García et al., 2024). Compared to the control group protocol, which did not significantly change mental fatigue (*p* = 0.44; Cohen’s d = 0.02), reaction time (*p* = 0.25; Cohen’s d = 0.01) and Stroop performance (*p* = 0.79; Cohen’s d = 0. 01), there was an increase in perceived mental fatigue (*p* < 0.001; Cohen’s d = 0.55) and a decrease in reaction time (*p* < 0.001; Cohen’s d = 0.37) and Stroop performance (*p* = 0.041; Cohen’s d = 0.15) (Díaz-García et al., 2024). Paddle specific accuracy of all strokes was also affected (*p* = 0.05) (Díaz-García et al., 2024).

## 4 Discussion

To the best of our knowledge, this is the first systematic review that synthesizes the mental health effects of padel and pickleball, two rapidly growing racket sports, in a joint analysis. From 71 studies, 14 (*n =* 1,403) met inclusion criteria, revealing distinct research focuses: padel studies emphasize performance-related mental health (e.g., anxiety, mental fatigue), while pickleball research highlights wellbeing benefits, particularly in older adults.

Padel’s performance focus shows that mental fatigue, exacerbated by consecutive games or tournaments, impairs reaction time and accuracy (Díaz-García et al., 2021a, Díaz-García et al., 2021b, Díaz-García et al., 2024; *p* = 0.05 for accuracy). Motivation may mitigate fatigue’s impact (McMorris, 2020), as seen in increased motivation during constrained matches (Díaz-García et al., 2021b). It has been recommended that coaches quantify the levels of mental fatigue and carefully manage mental load and fatigue during training, particularly in the sessions leading up to competition, to prevent excessive mental fatigue (Díaz-García et al., 2024). Anxiety, another key factor, varies by context—rising pre-competition and differing by gender, rank, and match outcome (Conde-Ripoll et al., 2024b; Conde-Ripoll et al., 2024a; Conde-Ripoll et al., 2023). Players aged 18 to 23 demonstrated higher self-confidence and lower somatic and cognitive anxiety compared to those aged 24 and over in padel specifically, confirming our findings on the role of age in psychological performance (Rodríguez-Cayetano et al., 2025). Notably, higher-level padel players exhibit greater self-confidence and lower somatic anxiety (Castillo-Rodriguez et al., 2022), though losing matches correlates with reduced self-confidence and increased cognitive anxiety, linked to forced errors (Conde-Ripoll et al., 2024b). However, a recent study found that international-level players reported lower levels of fatigue and self-confidence than national-level players, suggesting that global competition may uniquely impact self-confidence (Rodríguez-Cayetano et al., 2025). These findings align with broader sports psychology, where anxiety can impair or enhance performance depending on control (Raglin, 1992; Jones and Hanton, 2001).

In contrast, pickleball studies demonstrate significant wellbeing benefits, including improved life satisfaction (*p* < 0.05), happiness (r = 0.263, *p* < 0.001), and reduced depression (r = −0.23, *p* < 0.01) (Heo et al., 2018; Kim et al., 2021; Ryu et al., 2018). Social integration, particularly for women (*p* < 0.01, $\eta_{p}^{2}$ = 0.06), drives these outcomes (Sabe et al., 2022), supporting theories that social networks reduce depression (Reiner and Steinhoff, 2024). However, the COVID-19 pandemic highlighted vulnerabilities, with reduced pickleball participation linked to increased loneliness and lower mental health (Casper et al., 2021; *p* = 0.01). These findings underscore pickleball’s potential in mental health interventions for older adults (Heo et al., 2018), though its benefits may extend to younger players given its growing demographic (Mackie, 2025).

The divergent research focuses reflect the sports’ contexts: padel’s competitive emphasis versus pickleball’s recreational appeal. Yet, this leaves gaps—padel lacks wellbeing studies beyond emotional functioning, and pickleball under-explores performance-related mental health across ages. Gender imbalances also persist; only 22% of padel study participants were female versus 54% in pickleball, despite potential gender differences in anxiety and fatigue (Tian et al., 2022; Tornero-Aguilera et al., 2022). Age biases further limit generalizability, with padel studies skewing young (mean age ≤29) and pickleball focusing on those over 50, despite broader participation trends (Lecler, 2024; Mackie, 2025). Most studies involve elite or competitive players (*n =* 1,364), overrepresenting them relative to recreational players, who dominate participation (Lecler, 2024).

This systematic review provides valuable insights into the relationship between padel, pickleball and mental health. However, it is important to consider a number of limitations. First, the heterogeneity of the included studies in terms of methodology, sample size and outcome measures meant that a meta-analysis was not possible. Second, the smaller sample sizes limit the generalizability of the results. Third, there was an over reliance on self-reported data, lacking objective measures like cortisol for stress (Conde-Ripoll et al., 2024b). Fourth, there is a lack of randomized controlled trials and intervention designs. Further large scale and long-term randomized studies are needed to confirm/refute our findings. Fifth, the studies in padel typically included higher numbers of competitive and elite players, meaning that the experiences of recreational players, who make up the majority of participants in this sport, may not be fully captured. In contrast, the studies on pickleball focused predominantly on recreational players, particularly older adults. This may limit the applicability of the results. Sixth, the geographical concentration (pickleball in the USA, padel anxiety in Spain, exhaustion in Finland) suggests research silos (Denche-Zamorano et al., 2023). Seventh, excluding qualitative studies and searching only three databases (Medline, PsycINFO, Embase) may have missed relevant insights.

Future research should bridge these gaps by exploring wellbeing in padel and performance-related mental health (e.g., anxiety, fatigue) in pickleball. To achieve this, studies should include diverse populations, such as different age groups, genders, and skill levels, to ensure broader applicability of findings. Longitudinal and experimental designs are particularly needed to better understand the mechanisms linking padel and pickleball participation to mental health outcomes. Incorporating objective biomarkers, such as hormonal or autonomic measures, could further enhance our understanding of the physiological processes involved. As padel and pickleball participation grows globally (Fip, 2024), such studies could inform evidence-based strategies to maximize their potential as inclusive tools for mental health promotion.

## Data Availability

Publicly available datasets were analyzed in this study. This data can be found at: all data analyzed in this review are included in the original papers listed in the references.

## Supplementary material

SUPPLEMENTARY TABLE 1 Full details of the quality assessment of included studies using the Newcastle-Ottawa Scale (Wells et al., 2000), including individual item scores and overall ratings.

SUPPLEMENTARY TABLE 2 Full details of the quality assessment of the included study using the PEDro Scale (Page et al., 2021), including individual item scores and the overall rating.
