# Supplementary material for: Padel, pickleball and wellbeing: a systematic review
Source: Front Psychol. 2025 Jul 29;16:1614448. doi: 10.3389/fpsyg.2025.1614448 (PMC12341226; doi:10.3389/fpsyg.2025.1614448)
Supplement: SUPPLEMENTARY TABLE 1 — Full details of the quality assessment of included studies using the Newcastle-Ottawa Scale (Wells et al., 2000), including individual item scores and overall ratings. [file Data_Sheet_1.zip › Supplementary material/Detailed Quality of the included studies assessed by the PEDro-Scale (Page et al., 2021)..pdf]

Author	Data & Analysis	Randomization	Randomization	Comparability & Blinding	Comparability & Blinding	Comparability & Blinding	Comparability & Blinding	Data & Analysis	Data & Analysis	Data & Analysis	Data & Analysis	Total points
	Eligibility criteria specified	Random allocation	Concealed allocation	Groups similar at basline	Subject blinding	Therapist blinding	Assessor blinding	Less than 15% dropouts	Intention-to-trea analysis	Between-group statistical comparisions	Point measures and variaility data	
Diaz-Garcia et al., 2024		*		*	*					*	*	5
