# Supplementary material for: Padel, pickleball and wellbeing: a systematic review
Source: Front Psychol. 2025 Jul 29;16:1614448. doi: 10.3389/fpsyg.2025.1614448 (PMC12341226; doi:10.3389/fpsyg.2025.1614448)
Supplement: SUPPLEMENTARY TABLE 1 — Full details of the quality assessment of included studies using the Newcastle-Ottawa Scale (Wells et al., 2000), including individual item scores and overall ratings. [file Data_Sheet_1.zip › Supplementary material/Detailed Quality of the studies included assessed by the Newcastle-Ottawa Scale (Wells et al., 2000)..pdf]

| Author | Selection | Selection | Selection | Selection | Comparability | Comparability | Outcome | Outcome | Total points |
|---|---|---|---|---|---|---|---|---|---|
| | Representa-tiveness of the sample | Sample size | Non-respondents | Ascertainment of the exposure (risk factor) | Comparability: control for primary factor | Comparability: control for additional factor(s) | Assessment of Outcome | Statistical test | |
| (Casper et al., 2021) | * | * | * | ** | | | ** | | 7 |
| (Castillo-Rodriguez et al., 2022) | * | | | ** | | * | ** | | 6 |
| (Conde-Ripoll et al., 2023) | * | | | ** | | | ** | | 5 |
| (Conde-Ripoll et al., 2024b) | * | | * | ** | | | ** | | 6 |
| (Díaz-García, González-Ponce, et al., 2021) | * | | * | ** | | | ** | | 6 |
| (Heo et al., 2018) | * | | | ** | * | * | ** | | 7 |
| (Kim et al., 2021) | * | | * | ** | * | * | ** | | 8 |
| (Riffee et al., 2023) | * | | | ** | | | ** | | 5 |
| (Rodríguez-Cayetano et al., 2022) | * | | | ** | | | ** | | 5 |
| (Ryu et al., 2018) | * | | | ** | * | * | ** | | 7 |
| (Ryu et al., 2022) | * | * | * | ** | * | * | ** | | 9 |
| (Conde-Ripoll et al., 2024a) | | | * | ** | | | ** | | 5 |
| Diaz-Garcia et al., 2021a) | | * | | ** | | | ** | | 0 |
